# Cinders in the Eye

**Published:** 1856-09

**Authors:** 


### CINDERS IN THE EYE.

TRAVELLING by Rail is a fixed institution, and among the many millions who do it, there are not a few whose travelling for pleasure, turns out acutest pain, from locomotive cinders getting into the eye. They do not work out as readily as other foreign particles, because the rapid motion of the cars creates a current of air which gives the cinder such a momentum, that it plants itself in the body of the eye like a barbed arrow, and the usual resort of rubbing the eye only drives it in deeper.

NORTON CARROL. A fine name for a novel, and fitting would his life be, as material for an over strange tale. The contented and careless occupant of a one storied board house on the shore of the sea, his yacht and his angle rod, being the light of his eye and the delight of his heart, he floats along with the tide of life, all oblivious of position and fame and fortune and renown, yet the blood of Charles Carrol, of Carrolton, of the Clintons, of the Livingstons and other names of the old Knickerbocker stock, courses his veins. Born in the city of New York, a pupil of Columbia College, and reared amid all the luxuries which wealth and a family name could procure, abdicating all, long ago, he went down to the shores of the sea, and may any day be seen basking on Far Rockaway's beach; or in the stoop of his cabin, with rocking chair, cigar, and hoisted feet, and the latest novel, he dreams life's hours away.

But that life is not a vain one, for every mortal has his uses, whether of wood or hay or stubble or solid rock, or gold or diamond, in the great building of eternal ages, and he has communicated a fact, which is destined to save many an eye to Beauty, and many a tear from childhood's cheek, not for the hour or the day, but for all time to come. The sire's benevolence descended to his son, for little "Gabe" lost his life in the vain effort to save his cousin from drowning. Now, having roused the reader this hot August afternoon to the proper pitch of attention and remembrance, we empty our knowledge box before him.

Between the eye lid and the ball, introduce the bight of a horse or other strong hair, so as to include the spot where the particle appears to be, close the eye, and gently draw out the hair; the relief is said to be instantaneous, perfect and permanent.

